# Experimental and In Silico Approaches to Study Carboxylesterase Substrate Specificity

**DOI:** 10.3390/jox16010011

**Published:** 2026-01-12

**Authors:** Sergio R. Ribone, Mario Alfredo Quevedo

**Affiliations:** Unidad de Investigación y Desarrollo en Tecnología Farmacéutica (UNITEFA), Consejo Nacional de Investigaciones Científicas y Técnicas (CONICET), Departamento de Ciencias Farmacéuticas, Facultad de Ciencias Químicas, Universidad Nacional de Córdoba, Córdoba X5000HUA, Argentina; aquevedo@unc.edu.ar

**Keywords:** carboxylesterases, CES1, CES2, substrate, selectivity, HPLC, LC/MS, docking, MD simulation, QM/MM

## Abstract

Human carboxylesterases (CES) are enzymes that play a central role in the metabolism and biotransformation of diverse endogenous substances and xenobiotics. The two most relevant isoforms, CES1 and CES2, are crucial in clinical pharmacotherapy as they catalyze the hydrolysis of numerous approved drugs and prodrugs. Elucidating the structural basis of CES isoform substrate specificity is essential not only for understanding and anticipating the biological fate of administered drugs, but also for designing prodrugs with optimized site-specific bioactivation. Additionally, this knowledge is also important for the design of biomedically useful molecules such as subtype-targeted CES inhibitors and fluorescent probes. In this context, both experimental and computational methodologies have been used to explore the mechanistic and thermodynamic properties of CES-mediated catalysis. Experimental designs commonly employ recombinant CES or human tissue microsomes as enzyme sources, utilizing quantification methods such as spectrophotometry (UV and fluorescence) and mass spectrometry. Computational approaches fall into two categories: (1) modeling substrate: CES recognition and affinity (molecular docking, molecular dynamics simulation, and free-energy binding calculations), and (2) modeling substrate: CES reaction coordinates (hybrid QM/MM simulations). While experimental and theoretical approaches are highly synergistic in studying the catalytic properties of CES subtypes, they represent distinct technical and scientific fields. This review aims to provide an integrated discussion of the key concepts and the interplay between the most commonly used wet-lab and dry-lab strategies for investigating CES catalytic activity. We hope this report will serve as a concise resource for researchers exploring CES isoform specificity, enabling them to effectively utilize both experimental and computational methods.

## 1. Introduction

Human carboxylesterases (CES, EC 3.1.1.1) are enzymes belonging to the serine hydrolase family, responsible for the metabolism and biotransformation of diverse endogenous substances and xenobiotics containing ester, thioester, amide, carbonate, and carbamate moieties [1,2,3]. CES are classified based on amino acid sequence homology into five isoforms (CES1–CES5), among which CES1 and CES2 constitute the most clinically relevant isoforms [4,5,6].

**Figure 1:**
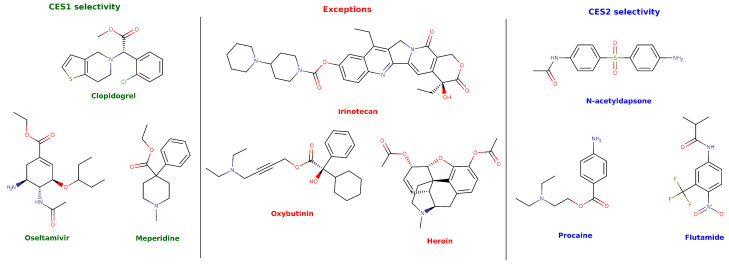
Molecular structures of substrates selectively hydrolyzed by CES1 or CES2, and exceptions to the general rule.

Although, CES1 and CES2 share 47% of protein sequence identity, they exhibit distinct substrate specificity profile and tissue distributions. CES1 is primarily expressed in the liver, whereas CES2 is predominantly found in the intestines [1,3,4,7]. It has been generally accepted that the substrate binding and hydrolysis selectivity of each isoform are primarily determined by the size of the acyl and alkyl moieties of the respective substrate’s molecular structures [4,6,7,8]. CES1 preferentially catalyzes the hydrolysis of substrates with smaller alkyl than acyl groups, as observed with drugs and prodrugs like clopidogrel, oseltamivir, and meperidine [2,8,9]. Conversely, CES2 preferentially hydrolyzes compounds with larger alkyl than acyl groups, including N-acetyldapsone, procaine, and flutamide (Figure 1) [1,3,8].

Despite this general trend, several exceptions to substrate selectivity have been reported. For instance, drugs and prodrugs like irinotecan, propanil, oxybutinin, and prolocaine, with different proportions of alkyl and acyl groups sizes, have been shown to be metabolized by both CES isoforms with similar efficiency (Figure 1) [8,10]. A widely reported exception is heroin, which is mainly metabolized by CES1 regardless of having a larger alkyl moiety relative to the acyl group (Figure 1) [11]. These and other contradictory reports strongly suggest that CES substrate selectivity is governed by subtle atomic-level details that drive catalytic efficiency. This is further supported by the differential CES-mediated biotransformation rates of *cis* and *trans* isomers of permethrin. Despite having identical acyl and alkyl group sizes, *trans*-permethrin is efficiently hydrolyzed by both CES isoforms, while *cis*-permethrin is hydrolyzed mainly by CES2 [12]. A similar phenomenon was reported in the study of the different hydrolysis patterns for the eight cypermethrin and four fenvalerate stereoisomers [13].

Understanding the structural determinants of substrate specificity among CES isoforms is crucial for optimizing drug therapy and developing targeted pharmacotherapeutic strategies, given the critical role of CES in physiological catalysis. However, the ability to predict the metabolic profile of a drug for each CES isoform remains largely unexplored. This gap in the field could be addressed in the near future through different machine learning approaches.

CES subtype-mediated bioactivation has enabled the design of prodrugs with optimized pharmacotherapeutic profiles. This approach prevents undesirable early biotransformation while enabling site-specific bioactivation by specific CES isoforms [2,14]. Currently, this strategy is being evaluated in a clinical trial examining CES1-expressing allogeneic neural stem cells combined with irinotecan for high-grade brain gliomas. The hypothesis is that intracranial administration of genetically modified neural stem cells enhances tumor sensitivity to irinotecan through site-specific bioactivation [15]. Similar targeting approaches using immortalized adipose-derived stem cells engineered to express carboxylesterase 2 have shown promise for castration-resistant prostate cancer treatment [16], demonstrating the therapeutic potential of both endogenous CES levels and stem cell-engineered enzyme delivery [17]. Amide-derived gemcitabine prodrugs exemplify the exploitation of endogenous CES levels for solid tumor treatment. These prodrugs are selectively bioactivated by CES2, which is overexpressed in certain cancer types [14].

CES are involved not only in drug hydrolysis but also play crucial roles in maintaining homeostasis through their participation in both physiological and pathological metabolic processes [18]. Beyond their established function in drug metabolism, CES enzymes influence lipid metabolism, detoxification pathways, and the regulation of endogenous substrates. These multifaceted roles have significant clinical implications, as variations in CES expression and activity can affect drug efficacy, safety, and individual patient responses. Looking ahead, advances in precision medicine may enable the development of tailored pharmacotherapy regimens that consider tissue-specific CES expression levels and substrate specificity of CES subtypes, ultimately improving therapeutic outcomes and minimizing adverse effects [19].

Specific inhibitors for CES1 and CES2 offer promising therapeutic potential for managing metabolic diseases [20,21]. For example, the CES1 inhibitor GR148672X has been explored for treating hypertriglyceridemia, obesity, and atherosclerosis [22]. However, CES1 inhibition can affect the metabolism of other drugs, resulting in clinically significant drug–drug interactions. Several clinical trials are investigating this effect, including one evaluating how concomitant cannabidiol treatment (a CES1 inhibitor) affects methylphenidate metabolism (a CES1 substrate) [23]. The clinical implications of approved drugs acting as CES inhibitors have been comprehensively reviewed elsewhere [6,24,25]. These considerations are especially important for drugs with narrow therapeutic windows, where CES inhibition can significantly impact drug efficacy and safety. Clinicians should be vigilant in recognizing potential drug–drug interactions mediated by CES inhibition, as these may alter therapeutic outcomes and increase the risk of adverse effects. While an in-depth discussion of these issues is beyond the scope of this work, it is essential for readers to remain aware of the critical role CES inhibitors play in clinical practice and patient management.

In the field of medical imaging, there is a growing interest in the development of fluorescent biological probes for selective detection of CES1 and CES2 activity [5,26,27,28,29]. Over recent years, the selective imaging of in vivo enzyme activity has emerged as a powerful method for the study of biological systems, due to its ability to provide real-time, noninvasive monitoring within living organisms [5,26,27,28,29]. In this context, the design of fluorescent probes that specifically target CES1 and CES2 offers a promising strategy for visualizing their activity in complex biological systems. An example is the use of CES2-mediated targeting potential, which is proposed as a prognostic biomarker for breast cancer because of the high CES2 expression levels in these cells [30].

Genetic polymorphisms in carboxylesterases (CES) can significantly influence the pharmacokinetics and pharmacodynamics of clinically approved drugs. Variations in CES genes may alter enzyme expression or activity, leading to differences in drug metabolism, efficacy, and safety among individuals. For example, polymorphisms in CES1 have been shown to affect the metabolism of the anticoagulant clopidogrel, resulting in variable therapeutic responses and increased risk of adverse cardiovascular events [31]. Similarly, CES1 genetic variants can impact the activation and clearance of methylphenidate, a drug used in the treatment of attention-deficit/hyperactivity disorder, influencing both efficacy and side effect profiles [32]. Another example is oseltamivir, an antiviral agent whose hydrolysis and activation are mediated by CES1; genetic differences in CES1 can lead to altered drug exposure and therapeutic outcomes [33]. Such genetic diversity can result in unpredictable therapeutic responses, increased risk of adverse effects, or drug toxicity, particularly for medications with narrow therapeutic windows or those extensively metabolized by CES. Recognizing and understanding CES genetic polymorphisms is therefore crucial for optimizing drug dosing, minimizing adverse reactions, and advancing personalized medicine in clinical practice. Although not discussed in detail here, readers should be aware of this clinically significant aspect and are encouraged to consult dedicated reviews for further information [34,35].

Enzymology has long been a foundational field for studying enzyme structure and function, advancing our understanding in intermediary metabolism, molecular biology, and cellular signaling [36]. Early enzymology focused primarily on experimental techniques analyzing both catalytic properties and molecular specificity of enzymes [36]. The first approach studies the thermodynamics and kinetics of the enzymatic reaction, measuring the free energy of reaction and activation. The second examines the specificity of different molecules for the enzymes, not only limited to the substrates, but also to any other molecule that satisfies the specificity criteria for the enzyme, such as potential inhibitors [36].

Due to enzyme complexity and the challenges of studying biomolecular reactions, many mechanisms remain unclear. Computational enzymology— the study of enzymes and their reaction mechanisms through molecular modeling and simulation [37,38]—uniquely enables investigation of biomolecular dynamic behavior and reactions at atomic resolution. This approach addresses unresolved issues by complementing and interpreting experimental findings [38,39,40]. Since the pioneering 1976 work of Warshel and Levitt (2013 Nobel laureates), computational enzymology has rapidly evolved through close collaboration between experimental and computational enzymologists, enhancing our ability to explain and interpret experimental data [38,39,40,41].

**Figure 2:**
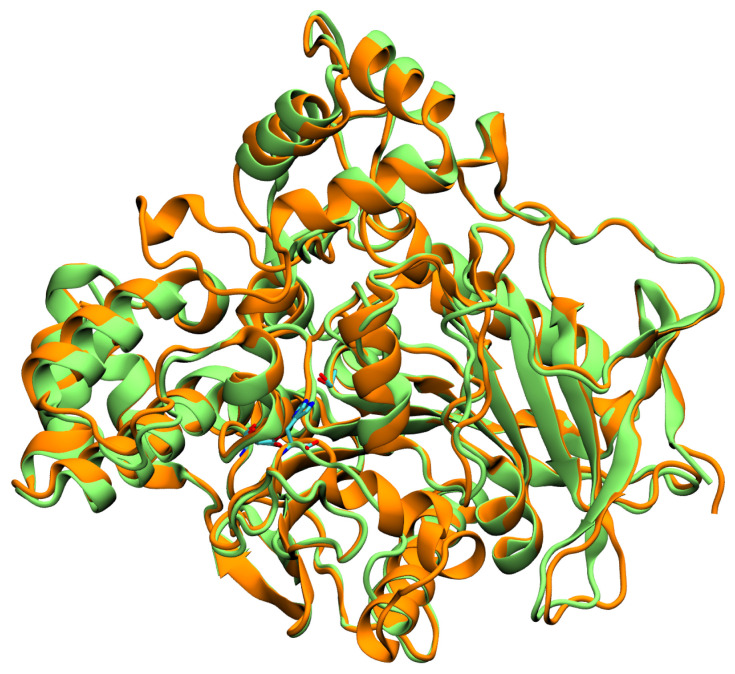
CES1 (orange) and CES2 (green) structural superimposition. The residues from the catalytic triad are indicated in licorice.

A major advantage for experimental and computational enzymology is the availability of crystallographic structures. Human CES crystallographic structures reveal three main functional domains: the catalytic domain, the α/β domain, and the regulatory domain [42,43]. In both CES isoforms, the catalytic site resides within the catalytic domain and contains the classical Ser-His-Glu triad characteristic of serine hydrolases (Ser221-His467-Glu354 and Ser228-His457-Glu345 for CES1 and CES2, respectively, as shown in Figure 2) [42,43]. Additionally, an oxyanion hole is present within the catalytic site, formed by residues Gly142-Gly143-Ala222 and Gly149-Ala150-Ala229 in CES1 and CES2, respectively [42,43].

**Figure 3:**
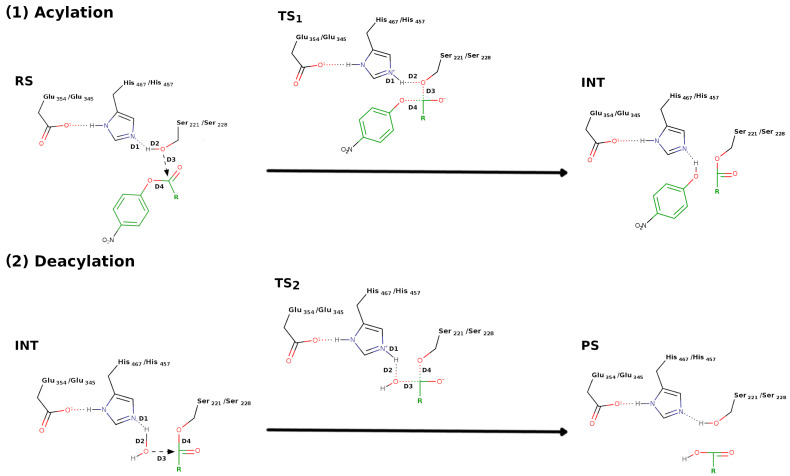
Acylation and deacylation reaction pathways for CES1- and CES2-catalyzed hydrolysis of the ester ligands. *p*-nitrophenyl ester derivatives were used as an example. Reactant state (RS), first transition state (TS1), intermediate (INT), second transition state (TS2), and product state (PS).

Another important piece of reported information is the general hydrolysis mechanism of esterases, such as CES, involving two consecutive reaction steps. First is the acylation step, where the carbonyl carbon of the substrate is attacked by the hydroxyl moiety from the catalytic serine, leading to the formation of an acylated serine intermediate and the release of the alkyl group of the substrate. Second is the deacylation reaction, where the carbonyl carbon of the acylated serine is attacked by the oxygen of a water molecule, yielding the caboxylic acid portion of the substrate and regenerating the free serine residue, thereby allowing a new catalytic cycle to begin (Figure 3) [3,42,44].

Building on the symbiotic relationship between experimental and computational enzymology, this review focuses on recent methodological advancements for studying substrate selectivity between the two major human carboxylesterase isoforms: CES1 and CES2. The first section discusses experimental enzymology approaches, covering enzyme sources and methodologies for calculating kinetic parameters. The second section examines molecular modeling techniques used to study substrate affinity and hydrolytic properties for both CES isoforms. Finally, the third section reviews current efforts combining experimental and computational studies to provide comprehensive analyses of CES substrate selectivity.

## 2. Part I: Experimental Enzymology

### 2.1. Kinetic Parameters

During enzymatic property studies, important kinetic parameters are determined using the fundamental Michaelis–Menten equation [36,45]. A first relevant kinetic parameter is the **Michaelis–Michaelis constant** (K_M_), which represents the substrate concentration at which the reaction rate reaches half of its maximum velocity (*V_max_*). K_M_ provides insight into the affinity of an enzyme for its substrate; a lower K_M_ value indicates higher affinity, while a higher K_M_ suggests lower affinity. Importantly, K_M_ is not simply the dissociation constant for the enzyme–substrate complex, but rather a composite parameter that reflects the rates of substrate binding. [2,13,36,44]. The second kinetic parameter is the **catalytic turnover**, determined by the catalytic constant (*k_cat_*), which represents the number of catalytic cycles that the enzyme can complete per unit of time when it is fully saturated with substrate. A higher *k_cat_* value signifies greater substrate turnover and more efficient metabolism [2,13,36,44].

The ratio between these kinetic parameters (*k_cat_* /K_M_) is the **specificity constant**, reflecting the ability of the enzyme to discriminate between different substrates. A higher *k_cat_*/K_M_ ratio indicates high substrate affinity (low K_M_ value) and high catalytic rate. Therefore, the specificity constant serves as a measure of **catalytic efficiency**, determining whether a given molecule is a good or poor substrate for the enzyme [36,44].

To obtain kinetic parameters that quantify substrate selectivity, experimental enzymology measures the progress of enzyme-catalyzed reactions. Like any other chemical reaction, enzyme-mediated substrate hydrolysis can be monitored by measuring either product formation or substrate consumption. Adequate detection methods for these processes are essential for successful enzyme assays [36]. Table 1 summarizes important information about enzyme sources and analytical methods used for the exploration of CES kinetic parameters on different reported substrates.

### 2.2. Enzyme Sources

This section outlines the different CES enzymatic sources used in reported experimental setups for calculating kinetic parameters. The two primary sources are ex vivo tissues, including purified human tissues and human microsomes, and pure recombinant enzymes (Table 1).

#### 2.2.1. Ex Vivo Tissue

Since the early 2000s, enzymatic studies of CES1 and CES2 substrate hydrolysis have relied on purified isoforms sourced from human liver tissue. Multiple studies have employed a standardized protocol involving tissue homogenization, centrifugation, and chromatographic separation of CES1 and CES2 isoforms [46,47,48]. Using this method, it was established that the produg irinotecan and related metabolites are primarily bioactivated by human CES2 isoform (Table 1) [46,47].

Another ex vivo source of CES is human tissue microsomes—small vesicles derived from fragmented cell membranes, primarily originating from endoplasmic reticulum. These human microsomes can be isolated through differential centrifugation or obtained commercially from biological supply companies. Human liver microsomes (HLMs) are commonly used as a standard enzyme source for metabolic stability assays due to their abundance of metabolizing enzymes. As mentioned in the introduction, CES1 is predominantly expressed in the liver, making HLMs a valuable source for studies of CES1 substrate selectivity. Conversely, human intestinal microsomes (HIMs) serve as a CES2 source, reflecting the tissue-specific distribution patterns described previously Using this comparative approach, researchers have demonstrated that the antiviral produgs oseltamivir and temocapril exhibit preferential activation by CES1 (HLMs) over CES2 (HIMs) [49,50].

#### 2.2.2. Recombinant Enzyme

The limitations of human tissue as an enzyme source soon highlighted the need for more purified CES isoforms to conduct accurate substrate selectivity assays. Morton and Potter addressed this challenge by developing a baculovirus-mediated expression system using *Spodoptera frugiperda* insect cells to produce recombinant CES1 and CES2 [64]. This breakthrough enabled precise enzymatic characterization of numerous substrates (Table 1). Notably, pyrethroid hydrolysis studies using these recombinant enzymes revealed distinct enantio- and diastereoselectivity profiles between CES isoforms and demonstrated the utility of fluorescent derivatives for evaluating CES1/CES2 hydrolysis activity and selectivity [13,54]. Subsequent investigations have expanded this approach to examine CES specificity for drugs of abuse (heroin, cocaine) [57] and other pharmaceutical drugs and prodrugs (Table 1) [8,58,59].

The commercial availability of recombinant CES enzymes from multiple suppliers has enabled extensive investigation of CES isoform selectivity across diverse substrates. Studies of the angiotensin-converting enzyme inhibitors enalapril and ramipril showed that both drugs were selectively hydrolyzed by CES1 [61]. Recombinant CES enzymes have proven instrumental in elucidating the predominant CES isoforms responsible for prodrug bioactivation, including sacubitril [62] and anordrin [63]. Additionally, recombinant enzymes have facilitated preclinical assessment of the bioactivation rates for rationally designed prodrugs, including atorvastatin [65,66], indomethacin [67,68], and haloperidol [69].

### 2.3. Analytical Methods Used in CES Enzymatic Assays

Accurate kinetic parameters are essential for successful enzymatic studies. Reliable analytical methods are therefore crucial for quantifying enzyme-catalyzed reaction progress [70]. This section describes the most commonly employed analytical techniques for kinetic parameter determination, highlighting their respective advantages and limitations.

#### 2.3.1. UV Spectrophotometry

**Figure 4:**
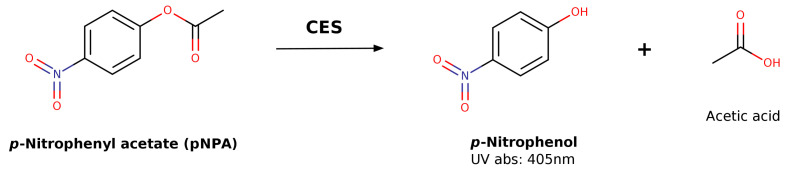
Hydrolysis of *p*-nitrophenyl acetate (pNPA) by CES to produce *p*-nitrophenol.

UV spectrophotometric methods have been widely employed for quantitative determination of total CES activity by measuring the absorbance of *p*-nitrophenol, produced through the hydrolysis of *p*-nitrophenyl acetate (pNPA), at 405 nm (Figure 4) [57,71,72]. This spectrophotometric approach has proven effective for the functional characterization of recombinant human CES expressed in *E. coli*, providing an alternative source of human enzyme [72].

The absorbance properties of *p*-nitrophenol have been used to evaluate the kinetic parameters associated with CES1 and CES2 subtype selectivity for various *p*-nitrophenyl ester derivatives [57]. The authors observed a correlation between the affinity constant (K_M_) and the calculated water/octanol partition coefficient (clogP) values, ranging from lower affinity constants and lipophilic values for *p*-nitrophenyl acetate (K_M_ 822 μM and 1.16, respectively) to higher values for *p*-nitrophenyl valerate (K_M_ 27 μM and 3.01, respectively), concluding that the affinity of the substrates for both CES isoforms is directly related to their lipophilicity properties [57]. Similarly, kinetic data for naphthyl ester derivatives were obtained by measuring the formation of naphthol at 230 nm [71].

The main advantages of this methodology are the easy access to UV spectrophotometers, the simplicity of the technique, and the fact that many substrates absorb in the UV spectrum. However, it has some limitations, such as the potential absorption interference between substrates and products, which complicates experiments in complex biological systems. Additionally, the method requires higher enzyme concentrations when performed in a UV cuvette with a total volume of 1 mL [72], while sensitivity may be compromised if the absorption coefficient of the measured species is insufficient.

In an attempt to solve several of the disadvantages associated with UV quantitation, chromatographic techniques able to separate substrate from products allowed a more precise quantification and higher reproducibility. This strategy employed HPLC-UV and it has been widely used to study the CES kinetic parameters of various therapeutic drugs and prodrugs, as most substrates exhibit absorbance in the ultraviolet spectrum [8,52,53,57,59,66,67,68,69,73]. To investigate substrate specificity among CES isoforms, a study was conducted on 13 compounds, including clopidogrel, clofibrate, oseltamivir, mycophenolate mofetil, procaine, and temocapril, among others (Table 1), using HPLC with specific conditions for each metabolite (e.g., mobile phase, column, and UV wavelength) [8]. This research group also applied the HPLC-UV method to study the hydrolysis kinetics of other drugs, such as flutamide [52,53], prilocaine, and lidocaine [59]. Based on the structural characteristics of these compounds, the authors proposed the already discussed general substrate selectivity pattern for CES: CES1 preferentially hydrolyzes ligands with smaller alkyl than acyl moieties (e.g., clofibrate, lidocaine, and temocapril, as shown in Figure 1), while CES2 favors those with larger alkyl than acyl groups (e.g., flutamide and procaine, as shown in Figure 1).

The biotransformation of the abuse drugs cocaine and heroin mediated by CES1 and CES2 has also been studied using HPLC-UV [57]. Cocaine hydrolysis was monitored by quantifying the formation of benzoylecgonine and benzoic acid at 235 nm. The results showed that cocaine was exclusively metabolized by CES2, producing benzoic acid and ecgonine methyl ester, while a second potential metabolic pathway forming benzoylecgonine and methanol was not detected [57]. In the case of heroin, its hydrolysis was assessed by monitoring the formation of 6-acetylmorphine at 235 nm. In this case, both CES isoforms were found to elicit hydrolysis, with CES1 exhibiting a catalytic efficiency twice as high as CES2 (*k_cat_*/K_M_ 9.7 and 5.9, respectively) [57]. This result represents an exception to the general CES1 substrate specificity, as was already mentioned in the introduction, as heroin has a larger sized alkyl group than acyl moiety in its structure.

Takahashi et al. have also reported several studies using HPLC-UV to investigate the CES subtype selectivity towards a diverse set of indomethacin-derived prodrugs [67,68,73]. Through the formation of indomethacin, monitored at 254 nm, and synthesizing prodrugs bearing a variety of alkyl moieties, the authors were able to investigate how different structural features influence the hydrolysis behavior of both CES isoforms [67,68,73]. Specifically, they examined the following: (1) the effect of the steric hindrance on the carbon adjacent to the carbonyl group, (2) the influence of electron density around the carbonyl group, and (3) the chiral recognition ability between CES1 and CES2. Overall, the results indicated that the indomethacin prodrugs were mainly hydrolyzed by CES1, because the drug structure represented the acyl group, which in all cases was larger than the corresponding alkyl group. However, prodrugs with aryl-containing alkyl moieties exhibited reduced or even no selectivity between CES isoforms, demonstrating that steric hindrance near the ester carbonyl carbon plays a crucial role in determining metabolic selectivity. This research group also conducted studies by synthesizing prodrug derivatives of haloperidol [69] and atorvastatin [66], leveraging the UV absorption properties of these drugs for detection using the HPLC method.

#### 2.3.2. Fluorescence Spectrophotometry

In contrast with the UV-based detection method, fluorescence-based analysis of enzymatic reactions provides both high detection selectivity and sensitivity. This quantitation strategy typically employs “*off–on*” fluorescent probes [74], representing molecules that initially exhibited little or no fluorescence (“*off*” state), but the hydrolyzed products released strong fluorescence in the presence of the corresponding enzyme (“*on*” state), enabling their detection. Several families of fluorescent probes have been specifically designed to quantify CES activity by monitoring changes in fluorescence intensity [13,55,75].

A very common strategy to quantify fluorescent probes, either for substrate selectivity measurement or for the screening of enzyme inhibitors, is using the 96-well flat-bottomed microtiter plates combined with a fluorescent spectrophotometer [76]. In addition to the selectivity and sensitivity of the fluorescent prove, this methodology enables high-throughput analysis with minimal material consumption, since a single analysis can be performed in every well using a total volume of 200 μL. By using 96 wells, it is feasible to design assay workflows to study in a single run one substrate at eight different concentrations in quadruplicate, considering both CES isoforms and a reference enzyme-free system. This methodology has been used to determine the kinetic parameters of pyrethroid-like substrates containing 6-methoxy-2-naphthaldehyde, in which the fluorescence was measured at an excitation wavelength of 330 nm and an emission wavelength of 465 nm [13]. In this study, it was observed that the steroisomeric centers of these derivatives presented a differential impact on hydrolysis by the two CES isoforms. Specifically, the presence of an (*R*)-enantiomer carbon adjacent to the ester carbonyl carbon resulted in a greater preference for CES2 hydrolysis than CES1 [13]. These findings highlight the importance of the three-dimensional disposition of the substrate groups within the catalytic site of the enzyme for CES hydrolysis selectivity.

**Figure 5:**
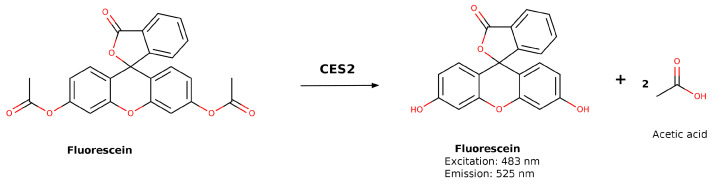
Hydrolysis of Fluorescein diacetate to produce Fluorescein as fluorescent substrate.

In another study, Fluorescein diacetate was used as a substrate to assess CES1 and CES2 selectivity. Hydrolysis of Fluorescein diacetate by the CES enzymes released Fluorescein, which was quantified at an excitation wavelength of 483 nm and emission wavelength of 525 nm. The kinetic results demonstrated that Fluorescein diacetate exhibited comparable K_M_ values for both CES1 and CES2 isoforms (4.31 and 4.82, respectively). However, the hydrolysis rate for CES2 was approximately 50 times higher than that for CES1 (14.6 and 0.213 μmol/mg/min, respectively), indicating a roughly 100-fold-greater k_cat_ value for CES2 compared to CES1. The results indicated that Fluorescein diacetate serves as a fluorogenic CES2-selective probe substrate for in vitro applications (Figure 5) [55].

**Figure 6:**
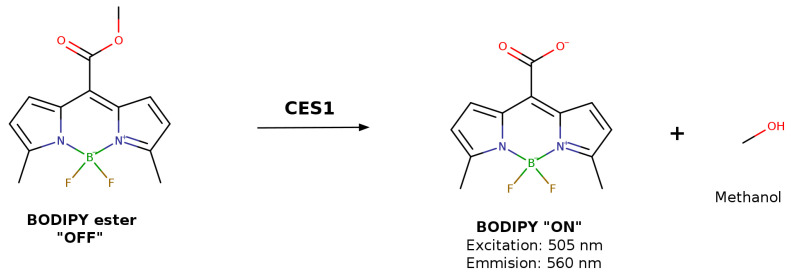
Hydrolysis of BODIPY ester to release BODIPY as fluorescent substrate.

The high fluorescence quantum yield and photochemical stability of Boron-dipyrromethene (BODIPY) dyes make them excellent candidates for this analytical methodology [77]. Consequently, a BODIPY ester was designed as a specific substrate for CES1. The acid product formed after CES hydrolysis was used to measure the kinetic parameters at excitation and emission wavelengths of 505 nm and 560 nm, respectively (Figure 6) [75]. Furthermore, this probe has been successfully employed for high-throughput screening of CES1 inhibitors using living cells as enzyme sources, demonstrating that these BODIPY derivative probes are practical tools for highly selective and sensitive sensing of CES1 activity in complex biological systems [75,78].

In these last two studies, the fluorescence was monitored by a fluorescent spectrophotometer after the corresponding separation of substrate and products through chromatographic methods (HPLC).

#### 2.3.3. Mass Spectrometry

In recent years, rapid advancements in mass spectrometry (MS)-based methods have made them suitable for efficient screening of enzymatic activity [79]. This technique is the most sensitive and accurate of all described analytical methods, as modern mass spectrometers can quantify very low molecular levels with high sensitivity and specificity. Furthermore, coupling MS detectors with chromatographic techniques, known as liquid chromatography–tandem mass spectrometry (LC-MS/MS), adds further specificity and quantitative power to this detection method. Regarding ionization sources, electrospray ionization (ESI) is the most widely used method in pharmaceutical analysis for nonvolatile and polar compounds, while atmospheric pressure chemical ionization (APCI) and atmospheric pressure photo-ionization (APPI) are preferred for nonpolar compounds [80]. Owing to the complementarity between ESI and APCI, the use of multimode ionization sources has been proposed for LC-MS analysis of samples containing analytes with a broad range of polarities and volatilities [80].

Ionization techniques are susceptible to signal suppression or enhancement depending on the sample matrix, a phenomenon known as matrix effects, which can compromise the reproducibility and accuracy of the method and lead to inadequate quantitation, particularly for researchers working with human tissue microsomes [80,81]. Various strategies can be employed to minimize matrix effects, including sample preparation methods and selecting the most appropriate ionization source prior to MS detection. The most common pretreatment approach is solid phase extraction (SPE), in which the majority of the tissue matrix is removed from the sample by elution through a solid phase cartridge [80,81]. Another strategy is the use of electron ionization (EI), as this technique completely converts the liquid from LC to the gas phase before ionization, resulting in analyte ionization that is less affected by matrix compounds or mobile phase composition [80,81].

Advancements in chromatographic column technology, such as hydrophilic interaction chromatography (HILIC), have further enhanced the possibility of studying enzyme kinetics in the context of in vivo metabolic pathways [82].

For example, LC-MS/MS has been used to investigate the metabolic pathways of diverse prodrugs, including capecitabine, a carbamate prodrug of 5-fluorouracil used in colorectal cancer treatment [83]. This study showed that this prodrug is hydrolyzed non-selectively by both CES1 and CES2, displaying very similar K_M_ (1.3 and 1.0 mM) and k_cat_ (18.8 and 13.5 min^−1^) values, demonstrating that substrates containing carbamate moieties can be metabolized by either isoform, regardless of the size of their acyl or alkyl moieties.

A prodrug of anordrin also showed similar hydrolysis profiles for both CES isoforms [63], with K_M_ values of 13.6 and 16.9 μM and hydrolysis rates (*V_max_*) of 1590 and 3321 pmol/min/mg for CES1 and CES2, respectively. This result is particularly interesting, since the anordrin alkyl moiety is significantly larger than the acyl group, which would preliminarily suggest CES2-selective hydrolysis [63]. Other prodrugs studied using LC-MS/MS include prasugrel, which was selectively bioactivated by CES2 [56], and sacubitril, selectively activated by CES1 [62].

**Figure 7:**
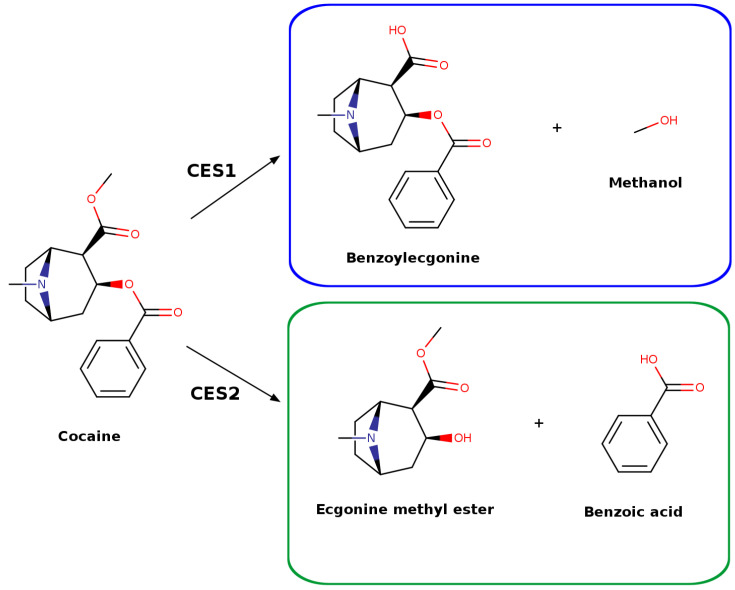
Different cocaine metabolic pathways from CES1 and CES2 experimental hydrolysis studies.

In a more recent study, cocaine hydrolysis as elicited by CES1 was re-examined using LC-MS/MS quantitation [42]. This study revealed that cocaine is also hydrolyzed by CES1, producing benzoylecgonine and methanol, contrasting with earlier HPLC-UV findings where this hydrolysis product was not detected (Figure 7) [57]. These results demonstrate that LC-MS/MS provides a more accurate analytical approach for studying CES substrate selectivity and hydrolysis compared to HPLC-UV.

In summary, both HPLC and LC-MS/MS are among the most sensitive and accurate analytical techniques, yet they share two significant disadvantages. First, these instruments are complex and not easily accessible, primarily due to their high purchase and maintenance costs. Second, operating them requires specialized training and experience. To achieve proper separation and ensure accurate results, various experimental conditions—such as solvent mixtures, temperature, run time, and pressure—must be systematically optimized by researchers or technicians skilled in these methodologies. Although tandem mass spectrometry is even more expensive and technically demanding, it offers unmatched specificity and the capability to simultaneously monitor multiple metabolites.

This section highlights the importance of experimental enzymology in studying CES substrate selectivity. The gathered evidence demonstrates that, despite the general structural rules established for substrate selectivity between CES1 and CES2, several exceptions and underlying structural properties remain unclear. Since CES binding and hydrolysis involve subtle intermolecular interactions, computational enzymology emerges as a crucial tool for analyzing CES substrate selectivity at the atomic level, as will be presented in the following section.

## 3. Part II: Computational Enzymology

### 3.1. Experimental CES Kinetic Parameter Databases

In bioinformatics, consistent kinetic data access through data management systems is essential for researchers retrieving enzymatic information from the vast biological literature published annually. Online databases have become invaluable tools providing high-quality structured information to support theoretical studies, which is the focus of this section. We begin our computational enzymology review by examining two widely used online databases containing human CES enzymatic parameters for various substrates.

#### 3.1.1. BRENDA Database

The BRaunschweig ENzyme DAtabase (BRENDA), established in 1987 at the German National Research Centre for Biotechnology, is the first and most comprehensive enzyme data collection compiled from the scientific literature [84]. With over 100,000 monthly users, the BRENDA website (www.brenda-enzymes.org, accessed on 20 November 2025) offers intuitive search capabilities through text queries (enzyme names, ligands, EC classes, inhibitors) or structure-based queries using drawn substrate/product structures [84]. As of October 2025, the search for CES (EC 3.1.1.1) information on the BRENDA website resulted in 2079 substrates/products and 67 natural substance entries. Among these data, 849 K_M_ values, 550 turnover numbers (*k_cat_*), and 232 *k_cat_*/K_M_ values (catalytic efficiency) can be retrieved from diverse bibliographic sources. In addition, the reaction diagrams and references associated with these substrates are provided.

However, two key limitations are noted using the website: (1) inability to filter information by enzyme organism origin, and (2) difficulty classifying kinetic data by CES isoforms. Modern computational enzymology requires local database interaction through structured data formats, yet BRENDA downloads are provided only as plain text files. To address this, the Python package BRENDApyrser (github.com/Robaina/BRENDApyrser, accessed on 21 November 2025) enables local parsing and manipulation of BRENDA data through structured queries and object-oriented methods. Such solutions make BRENDA a valuable resource for identifying kinetic parameters related to CES1 and CES2 substrate hydrolysis.

#### 3.1.2. SABIO-RK Database

SABIO-RK (sabiork.h-its.org/, accessed on 20 November 2025) is a manually curated database of biochemically relevant enzymatic reactions and their kinetic parameters, serving as a valuable resource for experimental and computational enzymology researchers [85]. Data are manually extracted from literature and stored in standardized formats, including reaction characteristics, biological sources, kinetic properties, and experimental conditions [85]. At the time of writing, searching “CES” in SABIO-RK returned 519 entries, fewer than BRENDA but with superior filtering capabilities. The advanced search feature enables filtering by organism (*Homo sapiens*: 168 entries) and enzyme source (recombinant CES: 82 entries). However, like BRENDA, SABIO-RK cannot filter kinetic parameters by CES isoforms. Despite containing less data than BRENDA, SABIO-RK offers better organization and more intuitive filtering options for retrieving relevant information.

These comprehensive repositories not only provide validated kinetic parameters for comparison and benchmarking but also offer detailed protocols that can guide experimental design and facilitate reproducibility across different laboratories. The integration of such databases with modern analytical techniques represents a powerful synergy that accelerates progress in CES enzymology research.

### 3.2. CES Structural Templates

Computational molecular modeling of substrate–enzyme complexes requires structural information for both components. While ligand structures are easily retrieved from biological databases mentioned previously, obtaining enzyme structures presents greater challenges due to their distribution across multiple sources and databases. This section discusses the structural availability of CES1 and CES2 enzymes.

#### 3.2.1. CES1 Structures

Since 2003, different crystallographic structures of CES1 have been reported (Table 2). The first published structures featured CES1 complexes with diverse ligands: drug metabolites (cocaine and heroin derivatives homatropine and naloxone methiodide) [11], therapeutic drugs (tacrine, tamoxifen, and mevastatin) [86,87], and endogenous substrates (cholate, taurocholate, and coenzyme A) [88]. Notably, the CES1–tamoxifen crystal structure revealed tacrine binding in four distinct modes within the catalytic site [86]. These findings highlight the promiscuous nature of CES1, demonstrating that its broad substrate hydrolysis capability stems from multiple ligand binding conformations [86].

Different types of covalent ligands have also been studied through crystallographic structures. Initial investigations focused on the organophosphorus nerve agents soman, tabun, and cyclosarin [89,90], while recent work explored serine-selective electrophilic warheads. Two crystal structures show that CES1 covalently binds to 2,2,2-trifluoroacetophenone derivatives at the catalytic serine (Table 2) [94]. Additionally, substrate-free CES1 structures (*apo* form) are available [91,92,93], with one (PDB: 5A7F) achieving the highest reported resolution for CES1 at 1.86 Å.

In order to perform molecular modeling studies of substrate selectivity between CES isoforms, the optimal approach uses high-resolution CES1 structures with non-covalent ligands or in substrate-free states. Crystal structures with covalent ligands should be avoided, as catalytic residues and binding site conformations may be biased toward covalent inhibitors rather than reflecting the intrinsic substrate recognition of the *apo* enzyme form.

#### 3.2.2. CES2 Structures

To date, the CES2 crystallographic structure remains unresolved due to N-glycan heterogeneity at the protein surface, which complicates production of non-glycosylated CES2 for crystallization studies [95]. Consequently, homology modeling is essential for obtaining three-dimensional CES2 structures to model substrate binding in the catalytic site. Various strategies have been reported for generating CES2 homology models, with the Swiss Institute of Bioinformatics server (www.expasy.org/, accessed on 5 September 2025) being most commonly used. Early approaches followed a two-step procedure: retrieving human CES2 amino acid sequences from Swiss-Prot, then submitting them to Swiss-Model [96] for automated homology modeling [43,97,98]. This methodology has since evolved, allowing for direct download of pre-existing CES2 models created by other researchers via Swiss-Model [44,99,100].

A second approach uses homology modeling software to generate CES2 structures. Several studies have employed the open-source *Modeller10.6* software [101] for CES2 homology modeling and subsequent molecular modeling studies [102,103].

In recent years, *AlphaFold*, a neural network-based methodology for predicting three-dimensional protein structures [104,105], has generated over 200 million protein structures since 2021, all freely available from the AlphaFold database (alphafold.ebi.ac.uk, accessed on 5 September 2025). Despite its accessibility and remarkable accuracy, to the best of our knowledge, the AlphaFold-predicted human CES2 structure has not yet been utilized for molecular modeling studies with substrates.

### 3.3. Molecular Modeling Methods

Complementing experimental enzymology studies, diverse molecular modeling methodologies enable atomic-level investigation of the structural basis determining kinetic parameters for CES substrates. This section reviews such approaches in two subsections: (1) modeling substrate–enzyme recognition to study affinity constants (K_M_) and (2) modeling substrate–enzyme reactivity to examine catalytic constants (*k_cat_*).

#### 3.3.1. Modeling of Substrate: Enzyme Recognition

CES substrate recognition studies typically begin with molecular docking [106] to identify substrate conformations yielding lowest-energy interactions within the catalytic binding site. Subsequently, CES–substrate complexes undergo molecular dynamics (MD) simulations to characterize dynamic behavior and stability in explicit aqueous environments at physiological temperature. Finally, free-energy interaction analyses are performed using MD trajectories. While few studies incorporate all three methods, most CES substrate investigations rely solely on molecular docking to identify optimal substrate conformations within the catalytic site.

Early molecular docking studies designed to explored the selectivity of CES1 and CES2 were conducted by the group of Vistolli et al. [43,107]. In these works, 40 known CES substrates were subjected to molecular docking protocols using two crystallographic structures of CES1 (PDB codes: 1MX9 and 1YAJ) and a homology modeling structure of CES2. Various scoring functions were used to calculate and correlate with the experimentally reported K_M_ values, thus enabling the development of predictive models of substrate affinity for both CES isoforms. Results revealed strong correlation between K_M_ and the calculated lipophilic interaction scores, highlighting the central role of hydrophobic interactions due to abundant apolar residues in the catalytic binding site [43,107].

**Figure 8:**
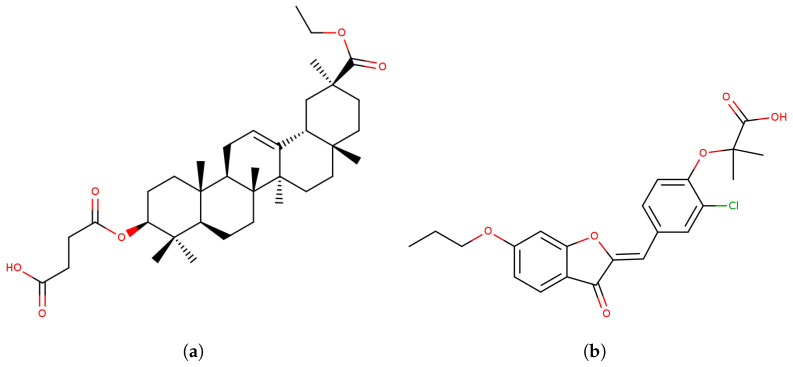
Molecular structures of CES2 selective inhibitors. (**a**) Glycyrrhetinic acid derivative. (**b**) Benzofuranone derivative.

Molecular docking and MD simulations have been employed to design selective human CES2 inhibitors. focusing on glycyrrhetinic acid and benzofuranone derivatives (Figure 8) [97,108]. In the first work, molecular docking was performed using the most active and selective glycyrrhetinic acid derivative against both CES isoforms to elucidate its 1000-fold preference for CES2 over CES1 [97]. Results showed greater hydrogen bonding with CES2 catalytic site residues than CES1, with strong interactions with catalytic residue Ser228 blocking CES2 substrate recognition and binding [97]. Similarly, molecular docking of the most selective benzofuranone derivative revealed more favorable interactions and binding contacts with CES2 than CES1 [108]. MD simulation and free-energy decomposition analyses of the CES2–inhibitor complex showed stability over 50 ns of simulation, with hydrophobic interactions playing major roles alongside hydrophilic residues like the catalytic Ser228 [108]. Both selective CES2 inhibitors present carboxylic acid groups in their molecular structures (Figure 4), suggesting that this structural feature could guide the design of novel, more selective CES2 inhibitors.

A recent study [44] performed molecular docking, MD simulation, and free-energy interaction decomposition analyses on two substrate families: five *p*-nitrophenyl ester derivatives [57] and two pyrethroid stereoisomers [13], using both CES1 and CES2. These results reveal a correlation between the experimental affinity constant (K_M_) and the total free energy of interaction (ΔG_Total_), with *p*-nitrophenyl acetate exhibiting both the lowest ΔG_Total_ and the lowest affinity constant, in contrast to *p*-nitrophenyl propionate, which displayed the highest ΔG_Total_ and the highest affinity constant.

Consistent with earlier findings, hydrophobic interactions were found to contribute substantially to the CES–ligand free energy of interaction, correlating well with experimental affinity constants (K_M_), as *p*-nitrophenyl ester substrates with higher affinity constants demonstrated a greater van der Waals (VDW) interaction component [44]. Additionally, the larger binding cavity volume of CES2 enabled one pyrethroid stereoisomer to adopt distinct interaction patterns, maintaining higher affinity (lower K_M_) than the other stereoisomer. All analyzed substrates formed optimal interaction patterns with respective CES binding site residues, positioning the carbonyl ester oxygen in the oxyanion hole near the catalytic serine, achieving maximum attainable affinity for each isoform [44].

#### 3.3.2. Modeling of Substrate: Enzyme Reactivity

The catalytic constant determination in enzyme-catalyzed hydrolytic reactions requires specialized computational methodologies to analyze the activation free energy associated with these processes. The most commonly employed approach for this purpose involves hybrid QM/MM simulations [109]. In this methodology, the substrate ligand and catalytic residues directly participating in the hydrolysis reaction are modeled using quantum mechanical (QM) calculations, while the remaining enzyme residues and surrounding environment are described using molecular mechanics (MM) force field approaches. The hydrolysis reaction mechanism is typically modeled using umbrella sampling methodologies [110], which represent enhanced sampling schemes specifically designed to overcome significant energy barriers by systematically restraining the molecular system to different predetermined points along a defined reaction coordinate in separate computational “*windows*”. By systematically combining and analyzing the results obtained from these individually biased simulations, this computational method generates an unbiased potential of mean force (PMF) or comprehensive free-energy profile [111], thereby providing researchers with a detailed and quantitative picture of the complete reaction pathway, including intermediate states, transition states, and their associated energetic profiles.

There are relatively few reported computational studies employing sophisticated hybrid QM/MM simulation methodologies to systematically explore human CES subtype selectivity patterns, likely attributable to the significantly greater computational complexity and technical demands of this advanced methodology compared with the more straightforward molecular modeling methodologies comprehensively described in the previous section. Given the critical importance and clinical relevance of human CES enzymes in the complex metabolic pathways of various abuse drugs, several comprehensive research investigations have systematically investigated the detailed molecular hydrolysis mechanisms of cocaine catalyzed by both CES1 and CES2 isoforms. The first pioneering study explored the complete hydrolysis mechanism of cocaine specifically catalyzed by CES1 using sophisticated hybrid QM/MM simulation approaches alongside parallel experimental determination and validation of the corresponding thermodynamic and kinetic parameters (K_M_ and *k_cat_*) [42]. The quantum mechanical region was carefully parameterized using the well-established semi-empirical SCC-DFTB computational method [112], while the molecular mechanics region was accurately described using the robust CHARMM27 force field parameters. The complex enzymatic reaction mechanism was systematically modeled using an advanced umbrella sampling computational approach, where the critical reaction coordinate (RC) was precisely defined as a mathematically linear combination of two essential covalent bond distances: one representing the bond formation process and one representing the simultaneous bond breaking process. These computational simulations indicated that CES1 effectively catalyzes the hydrolytic conversion of cocaine substrate to benzoylecgonine and methanol products (Figure 7) through a well-defined single-step acylation reaction mechanism followed by a subsequent single-step deacylation stage, with each individual step exhibiting a distinct and characteristic transition state (TS) structure. The combined free-energy profile analysis of both sequential reaction steps revealed that the acylation transition state represents the rate-limiting reaction step, associated with a calculated free-energy barrier of 20.1 kcal/mol. This computed theoretical result demonstrates remarkably close agreement with the experimentally determined free-energy barrier value, which was derived from the measured catalytic constant and found to be 21.5 kcal/mol, thereby conclusively demonstrating the exceptional accuracy and reliability of the hybrid QM/MM computational methodology employed throughout this study [42].

A second article reported a detailed computational study closely paralleling the previous investigation, systematically exploring the enzymatic hydrolysis mechanism of cocaine specifically catalyzed by CES2 to produce the alternative metabolic products ecgonine methyl ester and benzoic acid (Figure 7). In this complementary computational study, the quantum mechanical region was carefully parameterized utilizing the well-established semi-empirical PM6 computational method, while the molecular mechanics region was accurately modeled using the robust ff99SB force field parameters. An advanced umbrella sampling computational approach was again strategically employed to comprehensively follow and characterize the complete two-step hydrolysis reaction mechanism, wherein two critical covalent bond distances, one representing bond formation and one representing bond breaking, were systematically utilized as the defining reaction coordinates for the simulation protocol. The resulting two-dimensional potential of mean force (2D-PMF) profiles for the complete catalytic cycle demonstrated that each individual reaction stage proceeded through a well-defined tetrahedral intermediate molecular structure and involved two distinct transition state configurations. Among these identified transition states, TS_4_ (specifically associated with the formation of the benzoic acid product) was identified as the rate-limiting step governing cocaine hydrolysis catalyzed by CES2, displaying a calculated activation free-energy barrier of 19.5 kcal/mol. This comparatively lower energetic barrier relative to the corresponding CES1 mechanism is consistent with previous experimental findings reporting significantly higher turnover numbers for CES2 compared to CES1 under similar reaction conditions.

In the previous section, a comprehensive study involving two distinct families of substrates, *p*-nitrophenyl esters and pyrethroid stereoisomers, in a complex with both CES1 and CES2 enzymes was thoroughly described. As a follow-up investigation, two representative *p*-nitrophenyl ester derivatives and two specific pyrethroid stereoisomers were systematically subjected to hybrid QM/MM simulation methodologies to analyze their enzymatic hydrolysis mechanisms catalyzed by both CES isoforms [44]. The computational QM/MM simulation protocol followed was closely similar to the earlier pioneering study of cocaine hydrolysis with CES1 [42]; specifically, the quantum mechanical region was precisely parameterized using the advanced semi-empirical SCC-DFTB3 computational method, while the molecular mechanics region was accurately modeled using the ff14SB force field parameters as implemented in the Amber20 software package. As was the case for the previous example, this study strategically employed an advanced umbrella sampling approach; however, in this particular case, four critical covalent bond distances, two bonds undergoing formation and two bonds undergoing breaking (Figure 3), were systematically combined using a sophisticated linear combination of distances (LCOD) mathematical approach to precisely define the reaction coordinate (RC) for the simulation protocol. Across all modeled reactions, substrate hydrolysis catalyzed by both CES isoforms consistently proceeded through a well-defined concerted single-step acylation stage followed by another distinct single-step deacylation stage, with each individual stage involving one characteristic transition state (TS) structure. First, the results showed that for the hydrolysis reaction of the two *p*-nitrophenyl ester derivatives performed by both CES, the TS_2_ corresponding to the deacylation process was the rate-limiting reaction. Second, the free energies of activation involved in the hydrolysis of the *p*-nitrophenyl ester for both CES demonstrated that the two lowest *k_cat_* values (*p*-nitrophenyl trimethylacetate; *k_cat_* = 0.4 s^−1^ and 4.4 s^−1^ for CES1 and CES2, respectively) displayed the two highest TS_2_ free-energy activation barriers (12.7 kcal/mol and 10.6 kcal/mol, respectively). On the other hand, *p*-nitrophenyl propionate exhibits the two highest *k_cat_* (*k_cat_* = 7.9 s^−1^ and 52.5 s^−1^ for CES1 and CES2, respectively) and was found to exhibit the lowest TS_2_ energetic barriers (10.0 kcal/mol and 6.7 kcal/mol, respectively). Comprehensive analysis of the computational results clearly showed that the rate-limiting step for each individual substrate consistently corresponded to the transition state associated with the highest degree of molecular steric hindrance encountered during the course of the complete hydrolysis process, whether occurring during the acylation or deacylation stage [44]. The authors concluded that CES selectivity is not solely determined by the simple molecular size parameters of the alkyl or acyl functional groups of the substrate molecules, but instead arises from a significantly more complex mechanistic scenario governed by the initial three-dimensional conformation and orientation of the ligand molecule within the specific CES binding site environment [44].

Overall, all computational studies comprehensively discussed throughout this section consistently exhibited excellent correlations between experimentally determined catalytic constants (*k_cat_*) and the corresponding calculated activation free-energy values derived from the rate-limiting transition state reaction stages, thereby conclusively demonstrating the reliability and predictive accuracy of hybrid QM/MM computational methods. This remarkable agreement between theoretical calculations and experimental measurements was consistently observed despite the significant differences in the proposed enzymatic hydrolysis mechanisms across different substrate–enzyme systems, which involved either simplified two-transition-state or more complex four-transition-state structural arrangements during the complete catalytic process.

In summary, molecular docking, as the simplest computational technique, offers the advantages of very low computational cost and high simplicity and accessibility for conducting binding experiments. Its main limitation is that it only provides an initial substrate–CES complex conformation, without delivering accurate binding energy interaction data. In this context, MD simulations combined with free-energy interaction analyses yield more precise substrate binding results that can be compared with experimental affinity constants, though at a higher computational cost than molecular docking. Finally, hybrid QM/MM simulations present the highest computational cost among the described methods, often requiring access to high-performance computing clusters to perform all the necessary simulations. The primary advantage of this approach is the wealth of information it provides, from elucidating the enzymatic hydrolysis mechanism to determining free-energy activation barriers needed to correlate with experimental rate constants.

## 4. Part III: Combination of Experimental and Computational Enzymology

This final section of the article is devoted to studies that combined experimental and computational methodologies to investigate the hydrolysis properties of various substrates by both CES isoforms.

In the first reviewed study, the enzymatic hydrolysis of fenofibrate catalyzed by both CES1 and CES2 was systematically examined to definitively identify the primary enzyme responsible for its metabolic conversion in human biological systems [60]. The kinetic parameters were precisely determined by carefully measuring the formation rate of fenofibric acid (the primary hydrolytic metabolite of fenofibrate) by means of HPLC-UV analytical techniques in the presence of human liver microsomes and purified recombinant CES1 and CES2 enzymes. These experimental results demonstrated that the substrate affinity constant was marginally higher for CES1, but the most significant difference was observed in the catalytic constant values, with CES1 exhibiting a substantially faster enzymatic reaction rate compared to CES2 [60]. To provide computational support for these experimental findings, the authors performed molecular docking studies of fenofibrate within the active binding sites of both CES isoforms, discovering that fenofibrate consistently adopted a more thermodynamically favorable binding conformation and generated higher docking scores when positioned within the catalytic binding site of CES1 compared to CES2, results that were entirely consistent with the experimental substrate affinity measurements. Advanced hybrid QM/MM simulations were not performed or reported in this particular publication [60]. Overall, this integrated experimental and computational study provides valuable mechanistic insight into the molecular factors underlying the pronounced kinetic differences observed in fenofibrate hydrolysis between the two distinct CES isoforms.

**Figure 9:**
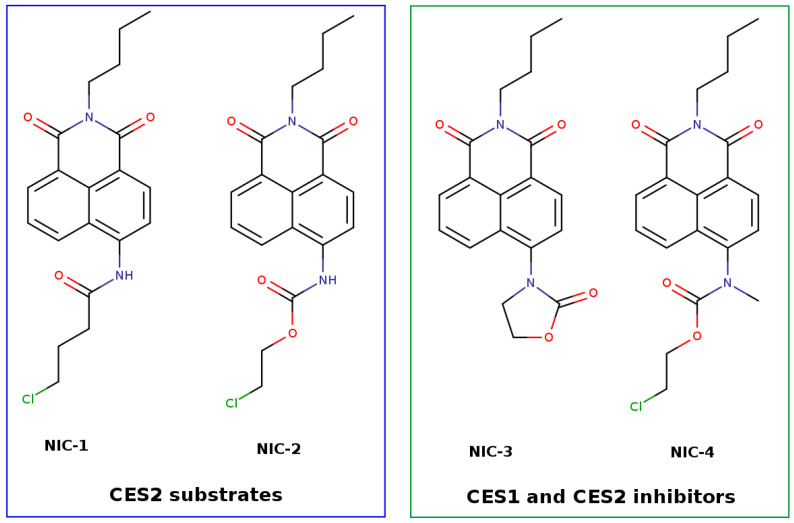
Structure of the fluorescent substrate derivatives of naphthalimide (NIC) [29].

The second study reported the design and CES selectivity of a family of four fluorescent substrates derived from a naphthalimide scaffold [29]. Enzymatic activity was monitored by measuring the emission intensity of the hydrolysis product at 520 nm (excitation 450 nm) after incubation with CES1 and CES2. Only the derivatives containing amide and carbamate moieties (NIC-1 and NIC-2, Figure 9) underwent specific hydrolysis in the presence of CES2. In contrast, the other two carbamates, in which the nitrogen was fully substituted with carbon atoms (NIC-3 and NIC-4, Figure 6), exhibited inhibitory effects of both CES isoforms.

To investigate the shift in activity from CES substrate to inhibitor, the binding properties of NIC-4 toward CES1 were analyzed using molecular modeling methods with CES1 and compared with the known substrate *p*-nitrophenyl acetate (pNPA) [29]. Molecular docking followed by MD simulations revealed that both NIC-4 and pNPA displayed similar interaction patterns with the residues of the CES1 binding site. In addition, a steered MD simulation was performed in which the structures and the binding site residues were parameterized with the SCC-DFTB method, while the remaining part of the enzyme was modeled applying parameters from the ff14SB force field. The reaction coordinate was defined as the distance between the catalytic serine oxygen and the carbonyl carbon of the substrate, representing the nucleophilic attack in the hydrolysis reaction. During the simulation of pNPA hydrolysis by CES1, the proton transfer from the catalytic serine to the nitrogen atom of the catalytic histidine occurred spontaneously, following the expected acylation mechanism with an estimated energetic barrier of 20 kcal/mol. In contrast, during the simulation of NIC-4 with CES1, the proton transfer did not occur, originating from the fact that the methyl group positioned near the potential nucleophilic center of NIC-4 (Figure 6) sterically blocked the transfer pathway, forcing the serine hydroxyl group to orient away from the histidine imidazole. The authors concluded that NIC-4 mimics the interaction pattern of the classic substrate to favor enzyme binding but hinders the necessary proton transfer process by methyl substitution of nitrogen in the carbamate, thus preventing the nucleophilic attack from happening [29].

This last work is a clear example of how integrating experimental and molecular modeling techniques is extremely complementary for exploring with higher accuracy the mechanism behind the substrate CES selectivity and also to assist in the design of selective CES inhibitors.

## 5. Conclusions

The main objective of this article is to show that traditional rules for explaining CES subtype substrate specificity need revision, favoring atomic-level analysis of structural features influencing catalytic site complementarity and steric factors. We emphasize the importance of integrating experimental techniques with advanced computational modeling, as this combination offers new insights into substrate selectivity between CES1 and CES2.

We aim to provide researchers with an overview of key tools in experimental enzymology and molecular modeling, promoting a shift from reductionist to holistic approaches that better capture enzyme–substrate complexity. The first section highlights how chromatographic methods paired with quantification techniques (UV spectrophotometry, fluorescence, and tandem mass spectrometry) form robust analytical frameworks for CES studies, each with distinct strengths and limitations for determining kinetic parameters. UV spectrophotometry is cost-effective but limited to chromophoric substrates, fluorescence methods enhance sensitivity for low-abundance products, and tandem mass spectrometry offers unmatched specificity and multiplexing.

Researchers are also encouraged to utilize enzymatic databases like BRENDA and SABIO-RK, which provide validated kinetic data and protocols, supporting reproducibility and accelerating CES research when integrated with modern analytics.

The second section reviews computational strategies—molecular docking, MD simulations, free-energy analyses, and hybrid QM/MM simulations—which yield accurate, experimentally consistent results for substrate–CES interactions. The benefits of combining experimental and computational approaches are evident in examples where this integration clarifies structural factors driving isoform selectivity.

Wider adoption of integrated methodologies will advance understanding of CES substrate selectivity, aiding the rational design of selective inhibitors, optimized prodrugs, and fluorescent probes. We hope this article serves as a valuable resource for ongoing research in this dynamic field.

## Figures and Tables

**Table 1:** Reported substrates with their respective enzymatic source and analytical methods used for the exploration of CES kinetic parameters.

Substrates	Enzymatic Sources			Analytical Method	References
	*Human Tissue*	*Human Microsomes*	*Recombinant Enzyme*		
Irinotecan	Liver	-	-	HPLC-FL	[46,47]
Methylphenidate	Liver	-	CES1/CES2	LC/MS	[48]
Oseltamivir	-	HLM/HIM	-	HPLC-UV	[8,49]
Temocapril	-	HLM/HIM	CES1/CES2	HPLC-UV	[8,50]
Aspirin	-	HLM/HIM	CES1/CES2	HPLC-UV	[50,51]
Clopidogrel	-	HLM/HIM	CES1/CES2	HPLC-UV	[8,51]
Flutamide	-	HLM/HIM	CES1/CES2	HPLC-UV	[52,53]
Pyrethroids	-	-	CES1/CES2	FL	[13,54]
Fluorescein diacetate	-	HLM/HIM	CES1/CES2	FL	[55]
Plasugrel	-	-	CES1/CES2	LC/MS	[56]
Heroin	-	-	CES1/CES2	HPLC-UV	[57]
Cocaine	-	-	CES1/CES2	HPLC-UV	[42,57]
Oxybutynin	-	HLM	CES1/CES2	LC/MS	[58]
Prilocaine	-	HLM	CES1/CES2	HPLC-UV	[59]
Lidocaine	-	HLM	CES1/CES2	HPLC-UV	[59]
Clofibrate	-	-	CES1/CES2	HPLC-UV	[8]
Fenofibrate	-	HLM/HIM	CES1/CES2	HPLC-UV	[8,60]
Imidapril	-	-	CES1/CES2	HPLC-UV	[8]
Enalapril	-	HLM	CES1/CES2	LC/MS	[61]
Sacubitril	Liver	-	CES1/CES2	LC/MS	[62]
Anordrin	-	HLM/HIM	CES1/CES2	LC/MS	[63]

HLM: Human liver microsome. HIM: human intestine microsome. FL: Fluorescence.

**Table 2:** Information related to CES1’s reported crystallographic structures.

Substrates	Classification	Resolution (Å)	PDB Code	References
Homatropine	M.L.	2.80	1MX5	[11]
Naloxone methiodide	M.L.	2.90	1MX9	[11]
Tacrine	Drug	2.40	1MX1	[86]
Tamoxifen	Drug	3.20	1YA4	[87]
Mevastatin	Drug	3.00	1YA8	[87]
Ethylacetate	M.L.	3.00	1YAH	[87]
Benzil	M.L.	3.20	1YAJ	[87]
Cholate/Palmitate	E.S.	3.00	2DQY	[88]
CoenzymeA	E.S.	2.00	2H7C	[88]
CoenzymeA/Palmitate	E.S.	2.80	2DQZ	[88]
Taurocholate	E.S.	3.20	2DR0	[88]
Soman	N.A.	2.70	2HRQ	[89]
Tabun	N.A.	2.70	2HRR	[89]
Cyclosarin	N.A.	3.10	3K9B	[90]
-	-	2.20	4AB1	[91]
-	-	1.86	5A7F	[92]
-	-	2.67	8EOR	[93]
F-3	C.I.	1.83	9KWL	[94]
F-4	C.I.	1.89	9KWM	[94]

M.L.: Metabolite ligand. E.S.: Endogenous substrate. N.A.: Nerve agent. C.I.: Covalent inhibitor.

## Data Availability

No new data were created or analyzed in this study.

## Abbreviations

The following abbreviations are used in this manuscript:

CES	Carboxylesterase
CES1	Carboxylesterase 1
CES2	Carboxylesterase 2
HLM	Human liver microsome
HIM	Human intestines microsome
UV	Ultraviolet
FL	Fluorescence
pNPA	*p*-nitrophenyl acetate
HPLC	High-performance liquid chromatography
BODIPY	Boron-dipyrromethene
LC/MS	Liquid chromatography–tandem mass spectrometry
MD	Molecular dynamics
QM/MM	Quantum mechanics/molecular mechanics
PMF	Potential of mean force
LCOD	Linear combination of distances
RC	Reaction coordinate
TS	Transition state
